# Periodontitis in Ischemic Stroke Patients: Case Definition Challenges of the New Classification Scheme (2018)

**DOI:** 10.3390/jcm11030520

**Published:** 2022-01-20

**Authors:** Cristina Andrada Costea, Ruxandra Christodorescu, Andrada Soancă, Alexandra Roman, Iulia Cristina Micu, Ștefan Ioan Stratul, Darian Rusu, Dora Maria Popescu, Aurel Popa-Wagner, Adriana Elena Bulboacă

**Affiliations:** 1 Department of Periodontology, Faculty of Dental Medicine, Iuliu Haţieganu University of Medicine and Pharmacy Cluj-Napoca, Victor Babeş St., No. 15, 400012 Cluj-Napoca, Romania; costea.cristinaandrada@yahoo.com (C.A.C.); andrapopovici@gmail.com (A.S.); veve_alexandra@yahoo.com (A.R.); 2 Institute of Cardiovascular Diseases Research Center, Victor Babeș University of Medicine and Pharmacy Timisoara, Bulevardul Revoluției, No. 12, 300024 Timisoara, Romania; ruxandra_christodorescu@yahoo.com; 3 Department of Periodontology, Anton Sculean Research Center of Periodontal and Peri-implant Diseases, Faculty of Dental Medicine, Victor Babeș University of Medicine and Pharmacy Timisoara, Bulevardul Revoluției din 1989, No. 9, 300230 Timisoara, Romania; sbs@online.ro (Ș.I.S.); rusu.darian@gmail.com (D.R.); 4 Department of Periodontology, Faculty of Dental Medicine, University of Medicine and Pharmacy, Petru Rareș St., No. 2, 200349 Craiova, Romania; dora.popescu@umfcv.ro; 5 Chair of Vascular Neurology and Dementia Center, University of Medicine, Essen, Hufeland St., No. 55, 45122 Essen, Germany; 6 Experimental Research Center in Normal and Pathological Aging (ARES), University of Medicine and Pharmacy, 200349 Craiova, Romania; 7 Department of Pathophysiology, Iuliu Hațieganu University of Medicine and Pharmacy, Victor Babeș St., No. 2–4, 400012 Cluj-Napoca, Romania; adriana.bulboaca@umfcluj.ro

**Keywords:** stroke, comorbidities, periodontal diagnosis, periodontitis, case definition

## Abstract

The identification of the associative relationships between ischemic stroke (IS) and risk factors such as advanced age and periodontitis is essential to design real screening protocols and to address them using primary and secondary preventive policies. This study primarily aimed to evaluate the diagnostic performance of the 2018 European Federation of Periodontology/American Academy of Periodontology (EFP/AAP) case definition in detecting periodontitis against the 2012 Centers for Disease Control and Prevention/American Academy of Periodontology (CDC/AAP) case definition in a group of IS patients. Secondarily, we report the periodontal status of IS patients and the associative relationship with respect to some risk factors. Patients with their first IS were assessed based on demographic data, medical, oral risk factors and periodontal parameters. The two case definitions were applied to identify the periodontitis burden. The agreement between the two case definition systems, as well as the misclassification ratio, were calculated. A total of 141 patients were included. According to the 2012 CDC/AAP and the 2018 EFP/AAP case definitions, a frequency of periodontitis of 98.5% and 97.8% based on two modalities of inclusion of cases in the severity groups, sensitivity values of 98.54% or 100%, and specificity values of 25% or 14.7% were calculated. Thus, the new case definition system has a higher capacity to detect periodontitis, especially the well-established forms.

## 1. Introduction

Stroke is the second most common cause of mortality worldwide [1], and it is the third leading cause of permanent acquired disability [2]. The findings on stroke burden from the Global Burden Disease Study 2013 reported that, in 2013, there were almost 25.7 million stroke survivors (71% with ischemic stroke (IS)), 6.5 million deaths from stroke (51% deaths due to IS), and 10.3 million new strokes (67% IS). The Global Burden Disease Study 2013 findings also indicated that two thirds of all strokes occurred among persons less than 70 years of age [3]. In addition, a significantly increasing trend was found for the evolution of the burden of stroke between 1990 and 2010, in terms of an increased absolute number of incidental strokes, deaths, and disability-adjusted life years loss [4]. Thus, it seems that primary stroke prevention is not effective enough, although it has long been considered feasible in practice [5]. The identification of the associative relationships between stroke and its risk factors is essential to design real screening protocols and to address them using primary and secondary preventive policies.

Since the first report on the epidemiological and etiopathogenetic association between periodontitis and IS [6], many studies have provided relevant information on this subject [7,8,9]. Several reviews reported a moderate association between periodontitis and IS [10,11,12,13], while others showed limited evidence sustaining this association [14]. An independent association was detected between periodontitis and IS risk, and particularly cardioembolic and thrombotic incidents, while regular dental care was shown to positively impact the reduction of the risk [15]. Hemorrhagic stroke does not have an infectious-inflammatory aetiology and has not been shown to be associated with periodontitis [6,9]. A risk ratio of 2.88 (95% confidence interval CI 1.53–5.41) of IS in periodontitis patients has been reported [10]. More recent information suggests that, globally, periodontitis patients have about twice greater chance of experiencing some type of stroke, as to having IS [16,17]. Therefore, it can be inferred that the reported association between IS and periodontitis [10,11,12,13,15,18] emphasizes the systemic implications of periodontitis [17].

Periodontitis is a chronic inflammatory disease driven by multiple interactions between the oral dysbiotic microbiota, the immune-inflammatory mechanisms of the host and a large palette of genetic, environmental and behavioral risk factors [19], which induce irreversible destruction of the periodontal structures and tooth-loss [20,21,22]. In IS patients, the more severe the periodontal destruction, the greater the risk of recurrent vascular episodes [23,24,25]. The pathogenetic substrates of the associative relationships between periodontitis and IS—a severe acute expression of atherosclerotic cardiovascular disease—are supported by the potential contribution of the periodontal infection to atherosclerosis-related inflammation through the bacterial invasion of the arterial walls, the systemic release of pro-inflammatory molecules and acute-phase reactants, such as the C-reactive protein [26], and pro-atherogenic effects of bacterial toxins [27,28]. It seems that atheroma plaques present a complex microbiota containing bacteria partially originating from the oral cavity [29,30]. Systemic antimicrobial treatments [23], as well as periodontal antimicrobial therapy [15,18] could, however, diminish the risk of IS. The inflammation process does not affect only the cerebral vessels. Several studies demonstrated that periodontitis is an independent risk factor for coronary artery disease [31].

Further studies are recommended to clarify the complex, albeit incompletely elucidated, pathogenic pathways between periodontitis and IS and to identify the relationship between the severity of the two diseases, the impact of the periodontal treatment and the influence of periodontitis on IS survival [16]. An important premise for further studies in this area of interest would be the use of an unanimously accepted case definition of the periodontal status in epidemiological investigations to allow comparisons between studies. Our review of scientific literature on epidemiological surveys indicates incomplete reports on periodontitis quantification and examination protocols. In addition, our findings reveal that the use of different periodontitis case definitions [32] generated inconsistencies in the reported prevalence rates, severity, and extent of periodontitis across different population groups, hampering data comparison [33,34,35].

The various classification systems employed to diagnose periodontitis in epidemiological surveys included their own case definitions and severity quantification scales [36,37,38]. The Armitage 1999 periodontal classification [39] has been extensively used in research and clinical activity, but its case definition was perfected only later by the Centers for Disease Control and Prevention (CDC) and the American Academy of Periodontology (AAP) (2012 CDC/AAP case definition) [36]. The 1999 classification was replaced by the 2018 European Federation of Periodontology/American Academy of Periodontology (EFP/AAP) classification of periodontal and peri-implant diseases and conditions, which released its own case definition system (2018 EFP/AAP case definition), and is based on more accurate diagnosis algorithms [22,40,41]. Both the 2012 CDC/AAP and the 2018 EFP/AAP case definition systems use a combination of parameters—clinical attachment loss (CAL) and probing depth (PD)—to define periodontitis, but the threshold values and their combinations are different. This can result in significant differences in periodontitis identification and prevalence values, creating confusion surrounding the associative relationships with risk factors or general diseases, thus hindering the direct comparison between studies [42].

This observation has been found particularly relevant in the context of assessing the impact of periodontitis in patients with various systemic diseases, given that obtaining population groups numerically adapted to the research purposes is extremely challenging, and the results of multiple studies with a uniform design, including a similar case-definition, need to be corroborated in order to increase the reliability of the results. The new 2018 EFP/AAP case definition system should, therefore, replace the 2012 CDC/AAP system in research to ensure the uniformity of reports and further comparisons. However, future studies should be carried out worldwide to assess the applicability of the 2018 EFP/AAP classification, and the way in which the modification of cut-off definition values impacts variation in disease prevalence against the former case definition system [43].

Based on this background, the primary aim of this study was to evaluate the diagnostic performance of the 2018 EFP/AAP case definition system in detecting periodontitis against the 2012 CDC/AAP case definition system, by assessing its sensitivity as well as its accuracy, in a group of IS patients. We hypothesized that the sensitivity of the 2018 EFP/AAP case definition system may exceed the value of 70%. Moreover, the secondary aim was to report on the periodontal status of the IS patients included in our sample and to assess the associative relationship between periodontitis and IS with respect to some behavioral and systemic risk factors.

## 2. Materials and Methods

### 2.1. Study Design and Population

A cohort prospective study was conducted at the Neurology Department of the Clinical Rehabilitation Hospital of Cluj-Napoca. The study respected the regulations provided by the Declaration of Helsinki on experiments implying human subjects, and received ethical approval from the Institutional Ethics Committee (No. 3/2018). The participants provided a written informed consent before physical examination and periodontal evaluation.

The patients in this study were recruited consecutively, daily, according to the hospital register and respecting the inclusion criteria. The patients were referred from other hospitals within the county, as well as from hospitals located in the north-central part of Romania. The inclusion criteria referred to patients at their first IS onset less than 6 weeks prior to the evaluation, providing the possibility to undergo a full-mouth examination, and to give informed consent. The participants in this study were subjected to a complete physical, neurological and cardiological examination. Antihypertensive medication or oral hypoglycemic drugs/insulin therapy were administrated for patients with hypertension or diabetes mellitus, respectively. The exclusion criteria referred to patients with a hemorrhagic stroke (confirmed by cranial computed tomography scan), recent myocardial infarction, acute infections treated with antibiotics, inflammatory diseases, degenerative brain disorders, oncologic diseases (in the past 5 years), immunosuppressant therapy, recent periodontal treatment (in the last year), and the presence of less than 6 teeth. Data, with respect to the above-mentioned diseases and conditions, were obtained from medical records. The report of this study followed the updated Standards for Reporting of Diagnostic Accuracy Studies (STARD) checklist of items [44].

The sample size estimation was based on the worst-case scenario provided in our previous study, including 93 IS patients that reported a 79.6% periodontitis frequency [45]. A frequency of 86.5% was obtained when edentulous patients were excluded from the analysis. Considering a periodontitis frequency rate of 80% among IS patients, a minimum sample size of 155 subjects, of which 124 subjects have periodontitis, is required to achieve a minimum power of 80% to detect a variation ranging from 0.70 to 0.90 of the sensitivity and specificity values, based on a target significance level of 0.05 [46].

### 2.2. Demographic and Systemic Medical Characteristics

Demographic data, such as age, gender, environment (urban/rural), body mass index (kg/m^2^), and behavioral characteristics (namely smoking, and alcohol consumption) were collected based on current recommendations. Smoking and alcohol use were defined based on the current frequency of use and, over time, quantity consumption, according to the National Center for Health Statistics criteria listed in the CDC glossary. Patients were divided into two categories (smoking and non-smoking, alcohol use and no alcohol use) according to the current smoking and drinking status, number of cigarettes consumed over the patient’s lifetime, and the number of drinks consumed per week and over the past year [47,48].

Blood pressure (systolic and diastolic blood pressure), and electrocardiogram were measured in all patients as input data to identify the presence of cardiac comorbidities. Blood samples were taken from all subjects included in this study, in the morning, after a 12-h fasting. Standard parameters were measured: basal glycemia, total cholesterol, low density lipoprotein cholesterol, and high-density lipoprotein cholesterol, and triglycerides. Arterial hypertension, ischemic cardiac myopathy, and type 1 and 2 diabetes mellitus were diagnosed following the current guidelines.

### 2.3. Periodontal Evaluation

All patients from the study group received a full-mouth periodontal examination, and a periodontal chart was completed for each patient. The experienced investigators performing the examinations were previously calibrated (C.A.C., A.R., A.S., I.C.M.). Before the onset of the study, they received written instructions on study design, periodontal evaluation and data collection protocols, and attended two training meetings supervised by a senior periodontist (Ș.I.S.). Moreover, the intraclass correlation coefficients were calculated: 4 subjects matching the inclusion criteria, but not involved in the study were evaluated twice, 24 h apart. Intraexaminer and interexaminer reproducibility values were 0.95 and 0.94, respectively.

The full-mouth periodontal examinations were performed in a standard environment (natural light) using standard methodology and equipment (dental mirror, 1 mm marking periodontal probe—UNC-15 periodontal probe, Hu-Friedy, Chicago, IL, USA). Six sites per tooth were evaluated for probing depth (PD), gingival recession (GR), and clinical attachment loss (CAL). PD, GR, and CAL were evaluated according to standard clinical definitions [49]. All probing measurements were rounded down to the nearest millimeter. Tooth mobility and posterior bite-collapse were recorded. The full-mouth Gingival Bleeding Index was calculated. The Gingival Bleeding Index was defined as the total number of sites with gingival bleeding on probing, divided by the total number of sites per mouth (four sites at each tooth) multiplied by 100 [50]. Oral hygiene was rated using the Oral Hygiene Score. Scraping was performed in 3 sites at each tooth and calculated as a percentage [51]. The number of missing teeth was also recorded.

### 2.4. Periodontitis Case Definitions

#### 2.4.1. 2012 AAP/CDC Periodontitis Case Definition

The 2012 AAP/CDC periodontitis case definition is generally considered the diagnostic reference standard due to its extensive application in research in association to currently in use 1999 Classification of Periodontal Conditions. Based on measures of CAL and PD at interproximal sites, periodontal status was defined as a four-level categorical variable [36]: (a) mild periodontitis: ≥2 interproximal sites with CAL ≥ 3 mm and ≥2 interproximal sites with PD ≥ 4 mm at ≥2 teeth or 1 site with PD ≥ 5 mm; (b) moderate periodontitis: ≥2 interproximal sites with CAL ≥4 mm at ≥2 teeth or at ≥2 teeth interproximal sites with PD ≥ 5 mm, also at ≥2 teeth; (c) severe periodontitis: ≥2 sites with CAL ≥ 6 mm at ≥2 teeth and ≥1 interproximal site with PD ≥ 5 mm; (d) gingivitis (previous mentioned criteria not met, Gingival Bleeding Index > 10% and PD ≤ 3 mm) plus healthy periodontal cases (none of the above criteria present). Periodontitis case definitions were applied by four experienced, highly trained periodontologists (A.R., Ș.I.S., I.C.M., P.D.M.) [45,52,53].

#### 2.4.2. 2018 EFP/AAP Periodontitis Case Definition

Periodontitis was diagnosed with reference to the EFP/AAP case definition recommended by the classification system presented at the 2018 World Workshop on the Classification of Periodontal and Peri-Implant Diseases and Conditions [22,38], as follows: presence of interdental CAL at two non-adjacent teeth, or presence of buccal or oral CAL ≥ 3 mm associated with PD > 3 mm. Following the identification of the periodontitis cases, periodontitis staging was established using a specified algorithm. Initially, the algorithm computed the stage based on the most severe detected CAL, with a CAL of 1–2 mm indicating stage I (mild destruction), CAL of 3–4 mm stage II (moderate) and CAL ≥ 5 mm stage III (severe). In addition to severity, some complexity elements were considered to account for potential stage increases. Shifting from stage III to stage IV occurred if high levels of tooth mobility and/or posterior bite collapse were present. The number of missing teeth was not considered due to incomplete data on the cause of tooth loss. Stage II patients were reclassified as Stages III–IV if either PD ≥ 6 mm or furcation involvement were found. The application of the above-mentioned algorithm generated a four-level categorical variable scale: (a) stage I (mild) periodontitis; (b) stage II (moderate) periodontitis; (c) stage III plus IV (severe) periodontitis; (d) gingivitis cases (CAL = 0 and Gingival Bleeding Index ≥ 10%) and healthy periodontal cases [38,41].

### 2.5. Data Analysis

Data curation, data analysis and graphical illustration was performed using Microsoft Excel Spreadsheet software (Microsoft 365), Medcalc version 15.8 (Ostend, Belgium) and SankeyMATIC (built on top of the Sankey library of D3.js, produced by Steve Bogart) [54].

Periodontitis frequency was calculated for both classification systems as the sum of the three severity entities reported to the total number of subjects (percentage). The periodontal status of the patients was defined as follows: healthy + gingivitis, mild, moderate and severe periodontitis, according to the 2012 CDC/AAP classification, and healthy + gingivitis, stage I, stage II and stage III + IV periodontitis, according to the 2018 EFP/AAP classification. To provide information on the characteristics of the group, the four-level periodontal categorical variables generated by both classification systems were regrouped into two categories for the statistical analysis, with a cut-off value CAL = 5 mm: severe forms and their equivalent stage III + IV cases were assigned to the “severe” category, whereas moderate (stage II), mild (stage I) periodontitis, healthy status + gingivitis were included in the “other” category.

Categorical data were reported as number of cases and percentages, while continuous data were reported as mean and standard deviation. The following statistical tests were applied to compare groups: Student’s t-test was used for independent samples and Chi-square with Yates’ continuity correction was used for small samples. A *p*-value < 0.05 was considered statistically significant and a confidence interval of α = 0.05 was estimated.

To assess the agreement between the 2018 EFP/AAP periodontal classification/case definition system and the 2012 CDC/AAP case definition system, the latter being considered as reference, sensitivity (Sn), specificity (Sp), positive predictive value (PPV), negative predictive value (NPV), and accuracy were calculated. For the diagnostic tests, severity classes were established and analyzed in two ways based on the frequency of the four-level periodontal variables provided by both case definition systems. Firstly, the cases were regrouped into “severe” and “other” categories, a procedure that was also used to provide information on characteristics of the group (cut-off value CAL = 5 mm). Secondly, moderate + severe (stage II + III/IV) cases were regrouped together (cut-off value CAL = 3 mm), while mild (stage I), health + gingivitis cases were included in the “other” category. This regrouping aimed to reflect the intensity of the local inflammatory burden, which increased with the severity of the periodontal disease. The overlap and redistribution of cases suggested by the two case definition systems were illustrated in a Sankey diagram. A misclassification ratio, in terms of over- and underestimation of the 2018 EFP/AAP was also calculated considering all diagnostic categories. No missing data were present.

## 3. Results

**Figure 1:**
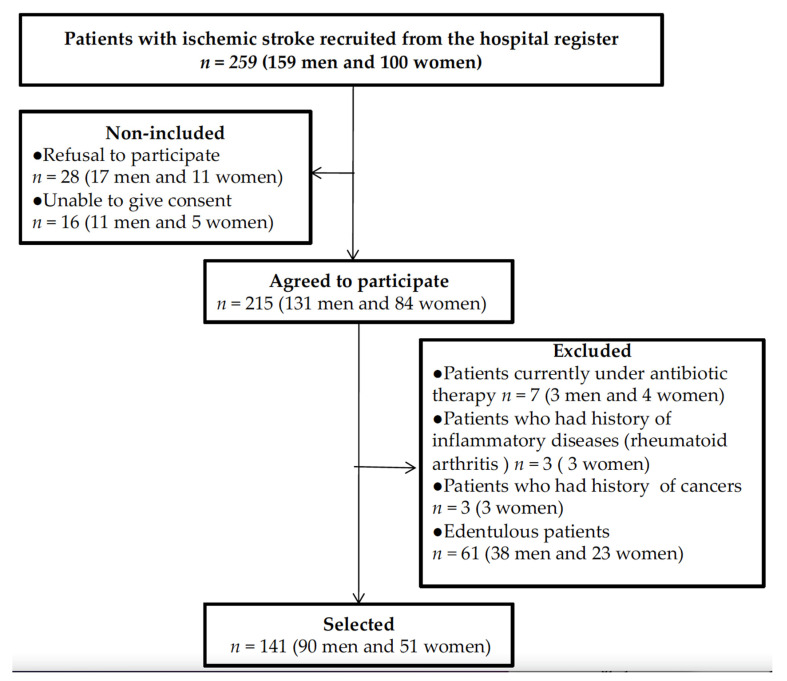
Flow chart of the patient selection.

From October 2018 to February 2020, a total of 259 participants aged between 24 and 88 years were selected for the study. Of the total group, 141 patients agreed to participate in the study and were eventually assessed for the statistical analysis (Figure 1).

### 3.1. Demographic, Behavioural, Medical and Periodontal Characteristics

Table 1 displays the distribution of demographic, behavioral, medical, and periodontal variables according to participants’ allocation to two periodontal conditions (severe periodontitis and other periodontal conditions) as identified by both case definition systems. When the 2012 CDC/AAP case definition system was considered, the Gingival Bleeding Index and Oral Hygiene Score indices were significantly higher in “severe” category patients than in the “other” category (*p* = 0.01 and *p* = 0, respectively). Our assessment of the 2018 EFP/AAP case definition revealed that the patients in the “severe” category were significantly older than those in the “other” category (*p* = 0.02). For both 2012 CDC/AAP and 2018 EFP/AAP case definition systems, low-density lipoprotein cholesterol values were significantly higher in the “other” category in comparison with the “severe” category (*p* = 0 and *p* = 0.03, respectively). No other significant differences in recorded parameters between the two considered periodontal categories were highlighted irrespective of the case definition system.

### 3.2. Periodontitis Frequency and Severity According to Both Classification Systems

According to the 2012 CDC/AAP and the 2018 EFP/AAP case definition systems, the total periodontitis frequency was 98.58% and 97.87%, respectively. The distribution of periodontitis severity and of the other periodontal conditions are shown in Table 2.

### 3.3. Diagnostic Performance

The study determined the Sn, Sp, PPV, and NPV, as well as the accuracy associated with both classification systems, based on two ways to establish severity (two cut-off severity values). The diagnostic performance measures are provided in Table 3. Diagnostic accuracy values indicate a relatively large proportion of correctly classified subjects with severe/medium disease or severe disease and a high agreement between the two classifications. When a CAL = 3 mm cut-off value was applied to distinguish between more severe periodontal conditions and milder or healthy ones, moderate and severe periodontitis were included in the same category; a 96.45% (CI 93.40–99.51) accuracy of the 2018 EFP/AAP case definition system was obtained. For a CAL = 5 mm cut-off, severe periodontitis cases were considered against all the other periodontal conditions; an accuracy of 79.43% (CI 72.76–86.11) was calculated.

Sn was calculated to obtain probability of receiving a positive test result in subjects with periodontal disease (true positive (TP) + false negative (FN)), more precisely to recognize subjects with periodontal disease. The high Sn values provided by this study indicate the important potential of the 2018 EFP/AAP case definition system to recognize severe forms of periodontitis. Sp values of our diagnostic test were calculated to examine the ability of the diagnostic test to identify subjects without the disease: subjects with mild periodontal pathologies or that were healthy (Sp = 25%), or subjects with periodontal conditions other than severe periodontal destruction (Sp = 14.7%). The PPV values suggest a relatively high probability that subjects included in the positive diagnosis group actually experienced severe or moderate periodontitis or severe periodontitis, respectively. For a CAL = 5 mm cut-off severity value, the NPV indicates a high probability that subjects with a negative diagnosis for this category do not experience severe disease.

**Figure 2:**
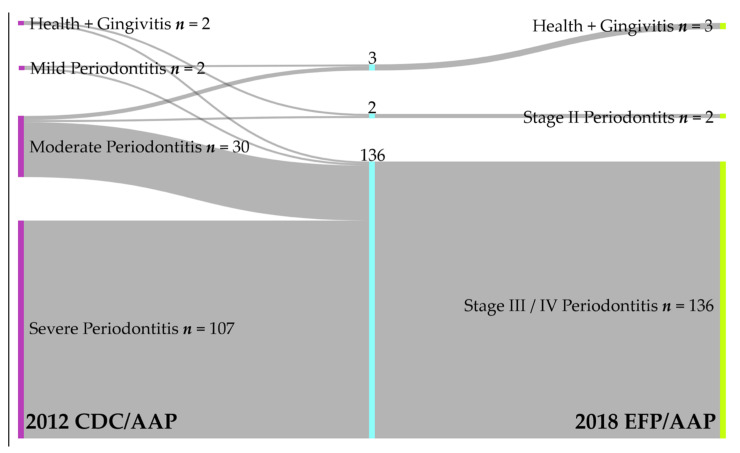
Sankey diagram. Comparison of cases classified according to the 2012 CDC/AAP case definitions as healthy + gingivitis, mild, moderate, and severe periodontitis (**left**) and the 2018 EFP/AAP case definitions with healthy + gingivitis, and stages I, II, III + IV) (**right**). From a total of 141 cases, an overlap of 108 cases (107 severe periodontitis and 1 moderate periodontitis) was calculated.

The study further assessed the way in which the identification of periodontitis patients based on the 1999 patient classification (2012 CDC/AAP case definition) was reflected by the 2018 EFP/AAP classification/case definition. When comparing the periodontal case definitions separately for the four-level variable scale (2012 EFP/AAP: health + gingivitis, mild, moderate and severe periodontitis vs. 2018 CDC/AAP: health + gingivitis, stage I, II, III + IV), a misclassification of 23.41% (*n* = 33) was calculated, mostly due to an overestimation (*n* = 30, 21.27%) of more severe conditions by the new case definition system. A comparison of the cases’ assignment using the 2012 CDC/AAP and the 2018 EFP/AAP case definition systems is provided in Figure 2 and Table A1. The diagram shows the overlap of the cases for the two classifications, as well as the misclassification of cases in terms of over- and underestimation, when the 2018 EFP/AAP case definition criteria were applied.

## 4. Discussion

A poor periodontal status of IS patients was recorded by both the 2012 CDC/AAP and the EFP/AAP classification systems, as revealed by the similarly high periodontitis burden: an overall periodontitis frequency of 98.5% and 97.8%, respectively, and a 75.89% and 96.45% frequency of severe cases, respectively. These results show a higher periodontitis burden compared with previous studies reporting a 79.6% [45] and 73.3% [55] periodontitis prevalence in stroke patients. However, it is important to acknowledge that previous reports were based on the 2012 CDC/AAP classification system [45] and the Community Periodontal Index for Treatment Needs (CPITN) criteria [55], respectively. Therefore, the overrepresentation of severe cases appears to be a particular feature of this specific study population.

Our study recorded differences between frequency values provided by the two case definition systems in relation to moderate forms (21.27%) identified by the 2012 CDC/AAP case definition system, which were almost entirely reclassified as severe forms (stage III + IV) by the 2018 EFP/AAP classification system, except for two cases (1.41% stage II or moderate forms). Thus, concerning the 2018 EFP/AAP case definition system, our results suggested an overestimation of periodontal involvement (21.27%), mostly due to the reclassification of some cases considered by the old case definition into more severe categories (Figure 2). The higher frequency of severe cases is in agreement with other studies reporting more severe conditions revealed by the 2018 EFP/AAP classification/case definition system that included mild forms of the former classification in stage II (moderate) of the actual 2018 EFP/AAP classification/case definition system. However, in contrast to our findings, previous results indicated a 100% agreement between classifications for the severe category [43].

Our study provides little information on healthy + gingivitis and mild periodontitis cases due to the very small number of patients included in these categories. As such, this further prevented us to draw firm conclusions on milder periodontal conditions based on the statistical analysis of the unbalanced study group and to generate the Sankey diagram (Figure 2). Nonetheless, the graphical representation highly suggests a redistribution of the cases based on the two classification systems. Although further research to evaluate the relationship between the severity of periodontitis and IS [16] is recommended, the present study could not address this issue since most of the patients were severely affected by periodontal disease. However, the current study should be seen as a model for future larger trials aimed at comparing the two classification systems in cases that do not belong to the severe ones.

Our study group was found to have very poor periodontal status, a category recorded by both classifications as moderate and severe periodontitis. An unfavorable periodontal condition in IS patients was previously reported by a pilot study published by our team [45], with 46.2% of the patients included in the moderate + severe category. The impact of these findings is highly significant since severe periodontal destructions are associated with increased mortality rate [56,57], as well as with cerebrovascular events [6]. Moreover, periodontal disease can double the overall risk for IS [16]; oral/dental infections were independently associated with the severity of atherosclerosis [58]. In our study group, there was a high frequency of hypertension, dyslipidaemia and diabetes, which are important risk factors for coronary artery disease. In addition, a high frequency of ischemic cardiomyopathy was also recorded, suggesting that vascular inflammation in patients with periodontitis was affecting not only cerebral vessels but also coronary arteries.

The 2018 EFP/AAP case definition system has been reported to have high Sn, implying that all periodontitis cases are identified due to a low CAL cut-off value of 1–2 [22], as compared to the 2012 CDC/AAP criteria (cut-off CAL ≥ 3 mm plus PD measurements) [36]. It may be inferred that the former 2012 case definition system did not consider mild destructions, which were automatically excluded from the periodontitis group based on CAL cut-off value. On the other hand, it is possible that incipient cases may be also overestimated by the current classification system due to errors intrinsic to the measurement of clinical periodontal parameters of ±1 mm [39,59] induced by many technical variables. Unlike the 2012 CDC/AAP case definition system, the new one not only lowers the CAL diagnostic threshold, but also considers mid-buccal and mid-oral periodontal measurements [41], which may increase the prevalence of periodontitis. However, this may lead to a miscalculation of the periodontitis case if the aetiology of buccal and oral CAL loss is incorrectly provided by the evaluator.

Relatively high Sn values were obtained, mostly when moderate and severe periodontitis cases were regrouped into a single category. In diagnostic tests, Sn is emphasized over Sp in some situations, such as very infectious or serious diseases. Periodontitis could be regarded as a serious disease because, when not screened and treated early, it induces irreversible destructions of the supporting tissues, as well as systemic consequences. A diagnostic system with high Sn and a high false-positive diagnosis rate is preferred because it detects incipient periodontal destruction allowing immediate treatment to arrest periodontitis progression [60,61]. On the other hand, the literature points to the importance of Sp for low prevalence conditions and a high cost of false-positive diagnosis [62], which is not the case for periodontitis.

A high PPV was calculated by the present study when severe and moderate periodontitis were considered separately. Both PPV and NPV are important parameters for already diagnosed individuals [43]. A moderate periodontitis, diagnosed according to the former classification system, would be referred and treated by dental practitioners; however, according to the 2018 classification system, the same condition would be categorized as severe, and the patient could benefit from a more specialized treatment.

Bothelho et al., determined Sn, Sp, accuracy, and precision after comparing the 2012 CDC/AAP and the 2018 EFP/AAP case definition systems and reported that the new 2018 EFP/AAP case definition outperforms the 2012 classification system regarding the diagnosis and staging of periodontitis on full-mouth partial recording protocols [63]. However, in our opinion, a return to the partial recording of periodontal parameters appears to be a step back both regarding the correctness of the examination and the possibility to identify the disease.

To our knowledge, no other agreement analysis between the two case definitions is currently available although other recent studies reported on periodontitis in relation to the two classification systems [64,65]. This point highlights one main strength of our study, which lies in the validity assessment of the 2018 EFP/AAP classification of periodontal conditions in a specific group of IS patients. Another strength of the study is the information input on the periodontitis status of IS patients based on the new 2018 EFP/AAP case definition system since no previous research is, to our knowledge, available in this context. Moreover, it seems that no reports on the use of the new classification in other systemically affected patients are available. However, our findings on the agreement between the two classifications may not be generalizable beyond the present setting.

In addition, other strong points of our study include the use of a gold-standard full-mouth examination associated with a six-site-per-tooth evaluation, as well as an appreciation of reference parameters by calibrated experienced operators and the assessment of a minimum number of six teeth. Clinical attachment loss is an indicator of past disease and a reliable diagnosis marker that accurately determines the intensity of periodontal tissue destruction in relation to a stable reference point—the cemento–enamel junction—but it also is the most reliable parameter for monitoring periodontal disease progression [32,66,67]. Pocket depth has been previously used as a specific indicator of local inflammation and current disease status [33,67,68], but used as a sole parameter it can generate errors in periodontitis identification due to the relative position of the gingival margin in relation to the cemento–enamel junction [33,35].

Based on the 2018 EFP/AAP case definition system, a set of parameters were identified as being significantly associated with the development of severe periodontitis. An increased age was associated with severity cases (*p* = 0.02), which is somehow expected as periodontitis destructions evolve over time [60]. The significantly lower low-density lipoprotein cholesterol values in the “severe” category against the “other” category (*p* < 0.05) could not sustain the idea of a proatherogenic lipid risk profile characterized by elevated serum levels of low-density lipoprotein, decreased levels of high-density lipoprotein, increased triglycerides, and increased serum levels of oxidized low-density lipoprotein in periodontitis patients [69].

Difficulty in collecting data is invariably linked to sample size, which could be regarded as a study limitation. This was a direct consequence of the pandemic conditions that prevented the recruitment of other IS patients. Moreover, we also must consider the important number of excluded edentulous persons (*n* = 61) which considerably diminished our study group.

The use of the new 2018 classification system/case definition as a universal classification system in further research would be a mandatory step to obtain relevant information on periodontitis epidemiology and impact, as well as to elaborate treatment strategies [70]. Moreover, in order to build on previous data and gain valuable insights in periodontitis research, it is also important to comparatively evaluate the accuracy of the different classification systems.

## 5. Conclusions

An extremely poor periodontal status in this group of ischemic stroke patients was identified by both case definition systems. An overestimation of periodontitis by the 2018 European Federation of Periodontology/American Academy of Periodontology case definition system as opposed to the 2012 Centers for Disease Control and Prevention/American Academy of Periodontology system was observed mostly for severe cases. The high sensitivity value underlines the capacity of the new case definition system to detect periodontitis, including mild periodontal destructions, which greatly favors early treatment.

## Figures and Tables

**Table 1:** Sample baseline characteristics based on both periodontitis case definitions.

Characteristic		2012 CDC/AAP Case Definition			2018 EFP/AAP Case Definition		
		Other (*n* = 34)	Severe (*n* = 107)	*p*-Value	Other (*n* = 5)	Severe (*n* = 136)	*p*-Value
***Demographic***							
Age *		61.88 (13.11)	63.79 (10.80)	0.40	52.00 (16.39)	63.74 (11.02)	0.02
Gender **	F	13 (25.5%)	38 (74.5%)	0.77 (Yates)	4 (7.8%)	47 (92.2%)	0.11 (Yates)
					
	M	21 (23.3%)	69 (76.7%)		1 (1.1%)	89 (98.9%)	
Environment **	R	17 (25.8%)	49 (74.2%)	0.67 (Yates)	1 (1.5%)	65 (98.5%)	0.44 (Yates)
	U	17 (22.7%)	58 (77.3%)		4 (5.3%)	71 (94.7%)	
BMI (kg/m^2^) *		26.99 (3.38)	26.69 (3.24)	0.75	26.66 (2.39)	26.76 (3.3)	0.96
***Behaviours***							
Smoking **	No	26 (26.3%)	73 (73.7%)	0.36 (Yates)	5 (5.1%)	94 (94.9%)	0.54 (Yates)
	Yes	8 (19%)	34 (81%)			42 (100%)	
Alcohol **	No	24 (23.8%)	77 (76.2%)	0.88 (Yates)	4 (4%)	97 (96%)	0.93 (Yates)
	Yes	10 (25%)	30 (75%)		1 (2.5%)	39 (97.5%)	
***Comorbidities***							
HTN **	No	10 (23.3%)	33 (76.7%)	0.87 (Yates)	2 (4.7%)	41 (95.3%)	0.98 (Yates)
	Yes	24 (24.5%)	74 (75.5%)		3 (3.1%)	95 (96.9%)	
Ischemic CM **	No	26 (27.4%)	69 (72.6%)	0.19 (Yates)	5 (5.3%)	90 (94.7%)	0.45 (Yates)
	Yes	8 (17.4%)	38 (82.6%)			46 (100%)	
DM ** (type 1 and 2)	No	22 (25%)	66 (75%)	0.75 (Yates)	4 (4.5%)	84 (95.5%)	0.72 (Yates)
	Yes	12 (22.6%)	41 (77.4%)		1 (1.9%)	52 (98.1%)	
***Biochemical parameters***							
CST (mg/dL) *		178.56 (48.29)	170.79 (104.03)	0.67	229.20 (60.69)	170.58 (94.06)	0.17
LDL-CST (mg/dL) *		104.76 (41.53)	89.74 (31.02)	0.03	139.40 (67.06)	91.67 (31.71)	0.00
HDL-CST (mg/dL) *		49.94 (11.78)	50.96 (15.11)	0.72	55.00 (11.38)	50.56 (14.45)	0.50
TG (mg/dL) *		123.59 (56.52)	125.72 (57.39)	0.85	86.40 (31.55)	126.63 (57.28)	0.12
Glycemia (BG) (mg/dL) *		114.88 (30.40)	124.75 (45.71)	0.24	101.8 (30.53)	123.13 (42.90)	0.27
***Periodontal parameters***							
GBI (%) *		33.54 (26.55)	47.85 (28.26)	0.01	36.05 (34.46)	44.71 (28.30)	0.51
OHS (%) *		60.96 (35.35)	79.69 (27.40)	0.00	59.00 (42.27)	75.76 (29.98)	0.23

* Continuous data are summarized as mean (standard deviation). ** Categorical data are summarized as the number of cases (percentages). *p*-values reflect the comparison between severe and other forms: Student’s test was used when the results are reported as means (standard deviation), and the Pearson’s chi-square test was used for categorical data. Abbreviations: F = female, M = male, R = rural, U = urban, BMI = body mass index, HTN = hypertension, CM = cardiomyopathy, DM = diabetes mellitus type 1 and 2, CST = total cholesterol, LDL-CST = low-density lipoprotein CST, HDL-CST = high-density lipoprotein CST, TG = triglycerides, BG = basal glycemia, GBI = Gingival Bleeding Index, OHS = Oral Hygiene Score.

**Table 2:** The frequency of periodontal conditions based on both case definition systems.

Periodontal Conditions	Frequency Values *n* (%)	
CDC/AAP—2018 EFP/AAP	CDC/AAP	2018 EFP/AAP
Health + Gingivitis	2 (1.42%)	3 (2.13%)
Mild Periodontitis—Stage I	2 (1.42%)	0
Moderate Periodontitis—Stage II	30 (21.28%)	2 (1.42%)
Severe Periodontitis—Stage III + IV	107 (75.89%)	136 (96.45%)
**Total**	**141**	**141**

**Table 3:** Diagnostic performance of the 2018 EFP/CDC classification system considering two cut-off variants.

	**2012 CDC/AAP**							
**2018 EFP/AAP**	**Yes**	**No**	**Total**	**Sn**	**Sp**	**PPV**	**NPV**	**Acc**
**Moderate + Severe category *n* (%)**				**98.54%**	**25%**	**97.82%**	**33.33%**	**96.45%**
Yes	TP 135 (97.82%)	FP 3 (2.17%)	138 (97.87%)					
No	FN 2 (66.66%)	TN 1 (33.33%)	3 (2.12%)					
Total	137 (97.16%)	4 (2.83%)	**141**					
**2018 EFP/AAP**	**2012 CDC/AAP**			**Sn**	**Sp**	**PPV**	**NPV**	**Acc**
**Severe category *n* (%)**				**98.54%**	**25%**	**97.82%**	**33.33%**	**96.45%**
Yes	TP 107 (78.7%)	FP 29 (21.3%)	136 (96.5%)					
No	FN 0 (0%)	TN 5 (100%)	5 (3.5%)					
Total	107 (75.9%)	34 (24.1%)	**141**					

Abbreviations: TP = True Positive FP = False Positive FN = False Negative TN = True Negative Sn = Sensitivity Sp = Specificity PPV = Positive Predictive Value NPV = Negative Predictive Value Acc = Accuracy.

## Data Availability

The datasets generated during the current study are available from the corresponding author on reasonable request.

## Appendix A

**Table A1:** Assignment of cases according to the 2012 CDC/AAP and the 2018 EFP/AAP case definition systems.

		2012 CDC/AAP				
		Health+ Gingivitis	Mild Periodontitis	Moderate Periodontitis	Severe Periodontitis	Total
**2018 EFP/AAP**	**Health+** **Gingivitis**		1	2		3
	**Stage I**					
	**Stage II**	1		1		2
	**Stage III/IV**	1	1	27	107	136
	**Total**	2	2	30	107	141
The table represents the number of cases assigned to each diagnostic category according to the two classification systems and the overlap as well as the misclassification of cases of the 2018 EFP/AAP classification system.						
